# Mexican traditional medicines for women’s reproductive health

**DOI:** 10.1038/s41598-023-29921-1

**Published:** 2023-02-16

**Authors:** Paulina Cabada-Aguirre, Alice M. López López, Keila C. Ostos Mendoza, Karen D. Garay Buenrostro, Diego A. Luna-Vital, Gail B. Mahady

**Affiliations:** 1 Department of Chemistry and Nanotechnology, Tecnológico de Monterrey, Ave Eugenio Garza Sada 2501 sur, Monterrey, Mexico; 2 Clinical Pharmacognosy Laboratory, Department of Pharmacy Practice, College of Pharmacy, PAHO/WHO Collaborating Centre for Traditional Medicine, University of Illinois at Chicago, Chicago, IL 60612 USA; 3 School of Medicine and Health Sciences, Tecnológico de Monterrey, Monterrey, N.L. México; 4 Tecnologico de Monterrey, The Institute for Obesity Research, Monterrey, N.L. Mexico

**Keywords:** Endocrine system and metabolic diseases, Health care, Health policy

## Abstract

Like China, Mexico has a traditional system of medicine dating back almost 5000 years that incorporates the healing practices of pre-Columbian civilizations, including the Maya and Aztec. Mexican Traditional Medicine (MTM) women depend on MTM practices and herbal medicines for their primary healthcare needs as limited access and high costs of Western medicine is a significant problem. The aims of this work were to determine the medicinal plants more commonly used in MTM for women’s reproductive health issues and assess the clinical data supporting their use. Data from multiple sources was compiled and information on plants commonly used by women in Mexico MTM for the treatment of fertility and menstruation issues, pregnancy, and menopause was analyzed. Analysis of the data show that 185 species of plants representing > 60 families were used for a wide range of reproductive health issues. Some of these plants have been used in MTM for fertility regulation in women of which 35 species were used as emmenagogues and abortifacients. Approximate 40 species were used for the symptoms of premenstrual syndrome, heavy menstrual bleeding, and dysmenorrhea. In terms of pregnancy, 35 species were used for postpartum care and to facilitate breastfeeding, 16 species were used as oxytocic agents to induce labor and speed birth, and six plant species were used to prevent miscarriage. Fourteen plant species were reported to treat infertility or promote fertility, and seven species were used to treat uterine prolapse. Three plants species were reported to treat menopause and two plants were used for osteoporosis. Analysis of the clinical data for commonly used medicinal plants showed some clinical support for the use of these plants in MTM. In Mexico, women use medicinal plants for almost every aspect of reproductive health. While some plants have clinical data, most medicinal plants used in MTM have no safety or efficacy data available and could serve as the basis of future investigations.

## Introduction

For thousands of years, traditional systems of medicine have played an important role in primary healthcare for populations worldwide. Similar to China’s traditional medicine (TCM) system, the ancient civilizations of Mexico, including the Maya, Inca and Aztecs had medical practices dating back ~ 5000 years^1,2^. Combining historic pre-Colombian Aztec, Mayan, and Incan healing traditions, with more recent African and Spanish-Catholic influences, Mexican Traditional Medicine (MTM) is a holistic system that emphasizes a balance between physical, emotional, and mental health as playing an important role in overall healing^1^. Interestingly, in addition to China, the MTM system shares many similarities with other Central and Latin American traditional healing practices^1,2^.

In MTM, women play a significant role in the overall healthcare of the family and the community^1,3^. For example, the *Señora/abuela* (female elder/grandmother) often plays a primary role in the initial familial dealings with sickness, and may be consulted for common illnesses, as well as marital/childrearing advice^3^. In addition, there are community healthcare workers called *Promotores de salud* (health promoters) that have some basic healthcare training, and serve as a link between the communities, Western healthcare clinics and mental health centers^3–5^. There are also several levels of specialty healthcare practitioners in MTM including herbal medicine specialists, massage therapists, midwives, as well as others^2,3,6^. The most common medical intervention in MTM is usually the herbal medicine practitioners, known as *herbolaria or yerbero/a*, who employ a wide range of herbal medicines to treat almost every disease^3,7^. The *partera* are the traditional midwives who participate in childbirth, but their role is not limited to medical attendance and assistance with birth^1^. The *partera* also uses herbal treatments, massages, and other treatments to aid the woman through the pregnancy, labor, and delivery^3^. Finally, the *curandero or curandera* play an important role in MTM to facilitate the patient’s recovery by restoring balance and order, thereby preserving the participant, family, community, and culture^7^. The *curandero* has an extensive knowledge of the therapies used for treating mental and physical illnesses; the use of herbs and medicinal plants, prayers, chants, and other rituals to restore an individual’s physical, emotional, and spiritual balance^3,7^. Thus, like other traditional systems of medicine, MTM practices holistic medicine that emphasizes restoration of balance between physical, emotional, and mental health.

Although Mexico has a registry of traditional healers, the exact number is currently unknown. This information is collected and maintained by the registries of the National Institute of Indigenous Peoples (Instituto Nacional de los Pueblos Indígenas – INPI, formerly Instituto Nacional Indigenista—INI) or the Mexican Social Security Institute (Instituto Mexicano del Seguro Social—IMSS)^8^. Interestingly, of all the MTM specialists, the percentage of women participating is quite high, and make up approximately 80% of midwives, 50% for herbalists, 50% bone-setters, 50% for healers (*curanderos*), 20% for snake healers (*culebreros*), 50% for shamans, 20% prayers and 40% for massage therapists (*sobadores*)^8^. The INPI is also responsible for providing training and certification for MTM practitioners and programs. Some MTM practices have certification and licensure, including acupuncture, herbal medicine, and massage therapy, and many MTM providers hold certifications for those specific MTM practices. Likewise, some curanderos have received education and certification as Naturopathic Physicians or other types of alternative care providers, which allows a level of practice consistency and some potential for credentialing and reimbursement for practices and procedures^9^. Certification generally occurs at the higher levels of MTM practice, and some herbalists and *sobadores* have completed education and certifications complementary to their practice^8^.

Medicinal plants have always played a central role in MTM, and anthropological and archaeological data suggest that human populations in Mexico have used medicinal plants for the treatment of disease for more than 5000 years^1,10–12^. Like other traditional systems of medicine, the use of these medicinal plants in MTM has been passed down by oral tradition, and many medicinal plants are still being used in the same manner as their historical use^1,3,10–12^. In Mexico, it is estimated that approximately 4500 species of plants (both native and introduced) are used by more than 52 different ethnic groups throughout Mexico, and many of these plants are recognized and used by ~ 90–92% of the general population^13^. Approximately 85% of healthcare professionals are knowledgeable about herbal medicines and ~ 75% recommend their use^3,13^. These data still show that traditional herbal medicines continue to play an important role in treating illness in rural communities, and are especially important for indigenous populations, despite access to modern health care clinics and prescription drugs. In fact, Rocha-Buelvas^14^ has suggested that within traditional medicine, herbal medicines play an essential role in the provision of culturally sensitive and accessible healthcare. Support for this hypothesis comes from data on Mexican immigrants in the USA who often use alternative and herbal medicines due to a lack of formal health insurance and their cultural beliefs. In a 2003 study, Brown & Pena^15^ reported 68.3% of pharmacists in Texas–Mexico border cities said they had encountered patients who used complementary and alternative medicines.

Currently, Mexico does not have a formal registry of traditionally used medicinal plants or herbal medications. However, many studies and medicinal plant collections have been performed by the INPI, the IMSS and the National Institute of Anthropology and History (INAH)^8^. In addition, some states maintain botanical and medicinal plant gardens. While traditional medicinal plants and medications are sold in specialized stores, quality control issues remain and there is little control over the production/collection of these products. The IMSS and BIENESTAR, formerly known as COPLAMAR (a government assistance program for rural and marginated communities) created a program to link traditional and Western medicine, that is operated by the same institutions. It was not until the late 1990s that the Ministry of Health established a classification of plants used in MTM to facilitate the scientifically review the safety and efficacy data for herbal medicines within its legal framework^16^. In 2013, the regulatory standard *Norma Oficial Mexicana* NOM-072-SSA1-2012 was enacted standardize these products, define legal usage, as well as the packaging of herbal remedies^17^. However, this regulatory law is not active in all parts of the country.

In this work, we have analyzed data from numerous databases, books, published scientific and medical works that reported the use of medicinal plants in MTM for the treatment of women’s reproductive health issues, including but not limited to menstruation, pregnancy, breast feeding, fertility, and menopause. The plant names were verified using official databases, corrected where needed, and then listed with uses and references. Experimental and clinical data are presented where available for the more commonly used medicinal plants in MTM.

## Methods

For this work, extensive literature searches were conducted over the period of September 2021 to March 2022 by three authors ALL, PCA and GBM. This included comprehensive electronic bibliographic databases (INFLIBNET, SCOPUS, PUBMED, UNAM, MEDLINE, NAPRALERT, and Google Scholar) and as well as from literature searches and analyses of data from published sources, including classical textbooks, books, and peer-reviewed journals from 1975 to March 2022 for medicinal herbs used in Mexico for women’s reproductive health issues and other associated disorders. Data was collected without language restrictions, using relevant keywords in both free text and Medical Subject Headings (MeSH terms) format. Terms for the searches of the scientific and medical literature included Mexican, traditional medicines, alternative medicines, breastfeeding, contraception, dysmenorrhea, emmenagogue, fertility regulation, pregnancy, menopause, osteoporosis, uterine prolapse and women’s reproductive health. The literature and studies published in English or Spanish were considered for this review. The Boolean connectors used included AND, OR and NOT. We also conducted searches of the alternative literature from UIC repositories, catalogues (UIC) for books, abstracts, and websites (OpenGray, GetNet International), and included conference proceedings from both national and international conferences. Four researchers (PCA, ALL, GBM and DL) independently extracted data from the studies using and disagreement between them was resolved by consensus with two other researchers (KM, KB). The botanical Latin binomials were verified using http://www.worldfloraonline.org (accessed January-March 2022, and plant names were corrected where needed after verification from published literature. Data from collected sources were collated into tables with uses and references by PAC, ALL, KM, KB). Data from clinical trials for some of the more commonly used medicinal plants associated with women’s health was also collected and analyzed by two researchers (GBM, DL).

## Results

### Clinical data for commonly used herbs in MTM for anxiety, dysmenorrhea, menopause, excessive menstrual bleeding, and PMS

Mexican women, particularly indigenous and those living in rural areas, use numerous medicinal plants as part of their overall health care. The purpose of this work was to investigate the medicinal plants used in MTM for the management of women’s reproductive health and provide a brief overview of some of the clinical and pharmacological support for these traditional uses for some of the more commonly used plants.

### Chamomile

Chamomile (*Matricaria chamomilla* L., Asteraceae) is a perennial flowering plant that is native to the Americas and is extensively used throughout Mexico and Central America^18–20^. Chamomile is one of the most widely used medicinal plants worldwide and is listed in the pharmacopoeia of at least 26 countries^20^. Traditionally, chamomile (also known as manzanilla) is used by women in Mexico and other countries as a mild sedative and to reduce anxiety^20,21^. In this work, the data show that chamomile was not only used to manage anxiety and depression, but also for the treatment of dysmenorrhea and excessive menstrual bleeding^12,22,23^. In support of these uses, extracts of chamomile and some of its chemical components have reported experimental and clinical anxiolytic and antidepressant activities^21,24^. In one clinical trial, Amsterdam et al.^21^ reported that chamomile treatment significantly reduced the mean general anxiety symptom scores as compared with a placebo after 8 weeks of treatment. In a follow-up study, the same group performed a randomized, double-blind, placebo-controlled, long-term safety and efficacy study of chamomile therapy for Generalized Anxiety Disorder (GAD)^25^. The study included outpatients with a primary diagnosis of moderate-to-severe GAD that were recruited from primary care clinics and local communities. The study results showed that participants exhibited a reduction in GAD symptoms and a reduced relapse rate when treated with chamomile^25^. Furthermore, the same group performed a randomized, double-blind, placebo-controlled trial of chamomile extract for relapse prevention of generalized anxiety disorder^24^. The study included 179 subjects who were ≥ 18 years old and had primary diagnosis based on the DSM IV-TR diagnosis of GAD. The subjects were further subcategorized into two diagnostic categories: GAD without comorbid depression (n = 100) and GAD with comorbid depression (n = 79). The treatment group received 1500 mg of chamomile extract daily for 8 weeks. The results showed that the chamomile extract significantly reduced clinical depression (p < 0.02) in addition to its anxiolytic activity in subjects with GAD and comorbid depression^24^. In several other clinical trials, treatment with a chamomile extract significantly improved sleep quality (p < 0.05) in the elderly and patients with insomnia, with no serious adverse events reported^26–28^. Thus, the traditional use of chamomile as a sedative and to reduce anxiety and improve sleep quality has been confirmed in clinical trials.

Furthermore, in support of its traditional use in MTM, chamomile was reported to reduce pain in dysmenorrhea and menstrual bleeding in clinical trials^29^. In a systematic review of the available data from seven clinical trials (n = 1033), two of the seven trials showed a significant reduction in the pain of primary dysmenorrhea (p < 0.01), while two studies showed a significant reduction in menstrual bleeding (p < 0.05), and three clinical trials showed significant improvements in both parameters after treatment with chamomile extract (p = 0.001)^29^. Thus, demonstrating that chamomile appears to be safe and efficacious for the management of primary dysmenorrhea and the reduction of excessive menstrual bleeding.

### Cinnamon

*Cinnamomum verum* J.Presl (Lauraceae, syn. *C. zeylandicum*) belongs to a genus containing approximately 200 species, of which, at least 19 have been identified in Mexico^30,31^. *Cinnamomum verum* is a species widely used in the traditional medicine of Mexico, although this species is thought to originate in Asia. In Mexico, cinnamon is used for the treatment of a wide variety of ailments, including respiratory diseases, abdominal cramps, indigestion, and nausea. In terms of women’s reproductive health, cinnamon has been used traditionally in MTM for the treatment of dysmenorrhea, regulating the menstrual cycle, as well as an abortive and oxytocic plant^23,32–34^.

Results from several clinical studies have shown that cinnamon administration significantly reduced the pain associated with menstruation^32,35^. One randomized, double-blind trial involving 76 female students with dysmenorrhea assessed the effects of cinnamon on pain and menstrual bleeding^35^. For inclusion in the study, the patients had to be 18–30 years old, with a regular menstrual cycle with primary dysmenorrhea, have no diagnosed chronic diseases, nausea or vomiting associated with primary dysmenorrhea, no pelvic inflammatory disease, tumors or fibroids, and a body mass index of 19–26. Exclusion criteria included intake of oral contraceptives, taking analgesics, and allergies to the medical or herbal extracts. The participants received placebo or a cinnamon product at a dose of 420 mg per day. The outcomes of the study showed significantly reduced menstrual bleeding in the cinnamon group as compared with the placebo group (p < 0.05 and p < 0.001, respectively). The mean pain severity score was also reduced in the cinnamon group as compared with placebo group. The mean severity of nausea and the frequencies of vomiting was also significantly decreased in the cinnamon group compared with the placebo group (p < 0.001, p < 0.05, respectively). No adverse effects were reported^35^.

An encapsulated cinnamon power was investigated for the treatment of polycystic ovary syndrome (PCOS) in a clinical trial involving 66 women with PCOS as diagnosed by the Rotterdam Criteria^36^. Administration of cinnamon was reported to restore the cyclicity and ovary morphology, as well as improve metabolic factors related to menstruation, specifically, it helps regulate insulin resistance. PCOS has been correlated to insulin resistance, which might be the reason why it helps with menstruation cyclicity. Outcomes measured included insulin resistance, anthropometric measurements, glucose and lipid profiles, and androgens. Each of the participants took medroxyprogesterone acetate 10 mg/day for the last 10 days of their menstrual cycles and, in the treatment group, women also received 1500 mg of cinnamon daily for 12 weeks. The results of this study showed that fasting insulin (p = 0.02) and insulin resistance (p = 0.014) were significantly reduced after 12 weeks of treatment with cinnamon as compared with the placebo group. A significant reduction in low-density lipoprotein in cinnamon group (p = 0.004) was also observed^36^. One meta-analysis of five clinical studies assessing the effects of cinnamon on PCOS did not find any significant effect on body weight or body mass index^37^. However, a significant decrease of fasting blood sugar and fasting insulin was observed as well as a reduction of serum level of LDL-C, total cholesterol, and triacylglycerol^37^. Interestingly, specific nutrients from food plants have also been reported to be important in the management of PCOS, and supplementation of inositol, combinations of vitamins D and E, probiotics, omega 3 fatty acids, and coenzyme Q10, with B vitamins improved glucose homeostasis and restored ovarian function and menstrual cyclicity^38,39^.

### Fennel

*Foeniculum vulgare* Mill (Apiaceae) is an aromatic plant native to the Mediterranean region of Southern Europe, that was introduced into Mexico and is now cultivated across the Sierra Madre^40^. In MTM, fennel has been used traditionally for the management of dysmenorrhea and heavy menstrual bleeding, to increase breast milk, as well as an analgesic and anti-inflammatory to manage menopausal symptoms^41–43^. Traditionally, fennel has also been used for the treatment of respiratory disorders, indigestion, PMS, osteoporosis, cardiovascular disease, as well as to increase breastmilk production and libido^43^. Clinical support for these uses is reported in a double blind, randomized clinical trial involving 60 postmenopausal women^44^. Treatment with a vaginal cream containing a fennel extract significantly (p < 0.05) reduced itching, dryness, pallor, and dyspareunia in women with vaginal atrophy as compared with the placebo group^44^. In another controlled clinical trial, 80 menopausal women (45–60 y) were randomized to receive treatment with encapsulated fennel seed powder (2 gr) or placebo capsules (2 gr) daily for 8 weeks^43^. Menopausal symptoms and sexual desire were evaluated using Kupperman index and Hurlbert index of sexual desire. Serum estradiol levels were measured at the beginning and at the end of the trial. The results of the study showed that menopausal symptoms were significantly (p < 0.05) reduced in the treatment group but there was no change in sexual desire^43^. In a triple-blind, placebo-controlled randomized trial involving 90 postmenopausal women aged 45 to 60 years^45^. The participants were included if they were 45 to 60 years old, married and in year 1–5 of the postmenopausal period (1 year after her last menstrual period). A minimum Menopause Rating Scale (MRS) questionnaire of 9 was also required, as well as no history of pathology, hormone replacement therapy or use of other herbal medicines, allergies to herbal medicine, sedative or anti-depressant drug use, addiction, or smoking. Treatment with the fennel extract significantly (p < 0.05) reduced the mean Menopausal Rating Scale score as compared with placebo. The results of the Friedman test showed significant differences between the mean score at baseline and the results at 4, 8, and 10 weeks (p < 0.001), indicating that the fennel extract was effective for the treatment of menopausal symptoms^45^.

In terms of premenstrual syndrome (PMS), clinical data suggests that oral administration of a fennel extract reduced the symptoms of PMS including stress, cluster and somatic symptoms, excitement, and depression^46,47^. One meta-analysis investigated the effects of fennel on pain associated with primary dysmenorrhea and compared these data with that of mefenamic acid^48^. Twelve clinical trials were included in the analysis, and the results showed that ingestion of fennel extracts significantly (p < 0.01) reduced the pain intensity of dysmenorrhea as compared with placebo. The effect of fennel versus mefenamic acid did not differ, suggesting that fennel supplements were as effective as mefenamic acid^48^. Thus, these results support the MTM use of fennel for dysmenorrhea.

### Rosemary

*Rosmarinus officinalis* L. (rosemary, Laminaceae) is native to the Mediterranean region of Europe and has been used in traditional systems of medicine as an antibacterial, antitumor, anticancer, antidiabetic, anti-inflammatory, antinociceptive, antioxidant and antithrombotic agent^49^. In MTM, rosemary has been traditionally used as an antispasmodic to treat dysmenorrhea, as well as a postnatal bath and to treat vaginal infections^22,50–53^. Lemonica et al.^54^ reported that rosemary extracts may have anti-implantation activities, but these extracts did not have abortive effects, nor did they impact the normal development of the embryo after implantation (no embryotoxic effects). Rosemary extracts have been used as antimicrobial agents against vaginal infections in pregnant women^55^. One in vitro study showed that essential oils of rosemary had antimicrobial activities against five Gram-positive and Gram-negative bacteria strains and two *Candida* strains obtained from pregnant women with vaginal infections^56^.

In one clinical trial, a rosemary extract was tested in a randomized double-blinded study involving 82 female students with primary dysmenorrhea^57^. To be included in the study, the participants had to have moderate dysmenorrhea with normal menstrual bleeding. Treatment with the extract significantly (p < 0.001) reduced the pain intensity score and the menstrual bleeding score, thus rosemary was effective for the treatment of primary dysmenorrhea, having similar activity to 250 mg of mefenamic acid capsules^57^. Several phytocompounds have been identified in rosemary extracts, such as caffeic acid, carnosic acid, chlorogenic acid, monomeric acid, oleanolic acid, rosmarinic acid, ursolic acid, alpha-pinene, camphor, carnosol, eucalyptol, rosmadial, rosmanol, rosmaquinones A and B, secohinokio, and derivatives of eugenol and luteolin. Eugenol, rosmanol, cirsimaritin and salvigenin appear to be some of the compounds in rosemary that are responsible for the antinociceptive effects, as they can act on the γ-aminobutyric acid (GABA-A) receptor^57^.

### Ginger

Ginger (*Zingiber officinale* Roscoe, Zingiberaceae) is a perennial herb with a subterranean, branched rhizome (root) that has a characteristic aromatic odor, a pungent and aromatic taste and are internally pale yellow to brown in color^58^. The plant is native to South-east Asia and is cultivated in the tropical regions in both the eastern and western hemispheres, and it is cultivated in Africa, China, India, and Jamaica, and India^58^. Ginger products widely used globally as teas, capsules, syrups, and dried rhizomes for the management of digestive diseases, inflammation and the nausea and vomiting of pregnancy. Oral administration of ginger-containing products has been used for the treatment of many gastrointestinal ailments including motion sickness, chemotherapy-induced nausea, post-surgical nausea and vomiting, and morning sickness^59–63^. In MTM, ginger is used for the management of nausea and vomiting in pregnancy. Clinical studies suggest that ginger may be useful for the management of morning sickness, as oral ginger reduced the severity of nausea and vomiting in some pregnant women^59–64^. The phenolic chemical constituents vary among the different forms of ginger and have a variety of pharmacological properties including antibacterial (including against *Helicobacter pylori*), antipyretic, analgesic, and anti-inflammatory activities, maybe explaining the effects of ginger on the gastrointestinal track. Viljoen et al.^64^ performed a meta-analysis and systematic review of the randomized controlled trials for ginger in the treatment of nausea and vomiting associated with pregnancy. Twelve randomized controlled clinical trials involving 1278 pregnant women were included in the review. The results of this analysis showed that ginger significantly (p = 0.0002) improved the symptoms of nausea at a dose of < 1500 mg/day as compared with placebo but did not reduce the incidence of vomiting. Ginger appears to be safe and did not increase the risk of spontaneous abortion compared with placebo^64^. Ginger was shown to be more effective than vitamin B6 in two studies, and its use was preferred over dimenhydrinate due to fewer adverse events. The overall safety of ginger use in pregnancy was confirmed by several of the trials that followed patients through childbirth. No significant adverse events were observed with ginger versus placebo. The limitations of these studies are due to the subjectivity in the measurement of nausea and the fact that the treatment times were short (one week at most), since most women experience longer period of nausea during their first trimester of pregnancy. The data suggest that ginger may have potential benefit for reducing nausea in pregnancy^64^.

### Herbal medicines used in MTM for women’s health with little or no clinical data

Beyond commonly used herbal medicines with experimental or clinical data, many of the plants used by women in MTM have little in the way of supporting pharmacological data. This is critically important as herbal medicines are commonly used by Mexican women during pregnancy (Tables 1 and 2)^22,51,65–71^. Alonso-Castro et al.^51^ reported a statistically significant (p < 0.05) use of both medicinal plants and allopathic medicines by Mexican women during the prenatal and postnatal periods. Herbal use during pregnancy was most often associated with prenatal symptoms including anxiety, nausea, fatigue, constipation, migraine, and common cold (p < 0.05)^51^. The results of this study suggested that 59.4% of pregnant women were self-medicating with herbal or allopathic medicines. Most recommendations for use of these herbs did not come from healthcare practitioners but from information provided by friends and family. Herbal products were most often obtained from pharmacies, local markets and supermarkets (45.2%), and the most common herbs used included *Matricaria chamomilla* L. (chamomile, 15.8%); *Citrus limon* (L.) (lemon, 9.1%), *Mentha piperita* (peppermint, 6.6%); *Arnica montana* L. (arnica, 4.8%); *Aloe vera* (aloe, 4.3%); and *Zingiber officinale* Roscoe (ginger, 3.9%), that were used to treat the symptoms of anxiety, backache, constipation, fatigue, infection, gastritis, and nausea (Table 2)^51^. Except for ginger and chamomile, most of these plants have no safety or efficacy data for use during pregnancy or delivery.

**Table 1:** Plants used in Mexican traditional medicine with emmenagogue and abortive properties, or used to treat menstrual cycle disorders.

Scientific name	Family	Common name	Emmenagogue and abortive properties	Menstrual cycle regulation	References
*Adiantum tricholepis* Fée	Pteridaceae	Fuzzy maidenhair	E, A		^53^
*Allium sativum* L.	Amaryllidaceae	Garlic	A		^23,33,72^
*Ananas comosus* (L.) Merr	Bromeliaceae	Pineable	A		^23,33,72^
*Anoda cristata* (L.) Schltdl	Malvaceae	Crested anoda		D M MH	^33^
*Aphelandra aurantiaca* (Scheidw.) Lindl	Acanthaceae	Fiery spike (espiga ardiente) Orange scarlet		D M MH	^23,33^
*Artemisia ludoviciana* Nutt	Compositae	White sagebrush		D	^50^
*Baccharis salicina* Torr. & A. Gray	Compositae	Sleep willow	E	D M MH	^23^
*Bignonia sp.*	Bignoniaceae	Begonia		M MH	^23,33^
*Bignonia potosina* (K.Schum. & Loes.) L.G.Lohmann	Bignoniaceae	*–*	A		^42^
*Brickellia secundiflora* (Lag.) A.Gray	Compositae	Jara Blanca		D M MH	^23,33^
*Cedrela odorata* L.	Meliaceae	Cedro	A		^34,42,73^
*Cestrum noctutnum* L.	Solanaceae	Huele de noche	A		^42,73^
*Cinnamomum verum* J.Presl	Lauraceae	Cinnamon	A	D, MH	^23,32–34,74^
*Citrus aurantiifolia* (Christm.) Swingle	Rutaceae	Key lime	E A	D	^33^
*Citrus* × *aurantium* L.	Rutaceae	Bitter orange	A	D	^12,23^
*Citrus limon* (L.) Osbeck	Rutaceae	Lemon	E A	D	^23^
*Columnea schiedeana* Schltdl	Gesneriaceae	Mazorquita		M	^23,33^
*Coriandrum sativum* L.	Lauraceae	Coriander	A		^23^
*Cymbopogon citratus* (DC.) Stapf	Poaceae	Lemongrass	E		^42^
*Desmodium incanum* DC.	Fabaceae	Zarzabacoa comun	A		^22^
*Dodonaea viscosa* (L.) Jacq	Sapindaceae	Native hop bush		M MH	^23,33^
*Dysphania ambrosioides* (L.) Mosyakin & Clemants	Amaranthaceae	Epazote	E A		^23,33,73^
*Epiphyllum crenatum* (Lindl.) Don	Cactaceae	Climbing orchid cactus	A		^22^
*Foeniculum vulgare* Mill.	Apiaceae	Fern		D, MH	^42,73^
*Galium mexicanum* Kunth.	Rubiaceae	Pegarropa	A	M MH PM	^23,33,42,74^
*Gaultheria acuminata* Schltdl. & Cham	Ericaceae	Wintergreen		D M MH	^23,33^
*Gossypium barbadense* L.	Malvaceae	Cotton	E, A		^42^
*Hamelia patens* Jacq.	Rubiaceae	Scarlet bush	A	MH	^22,34,70,72^
*Hybanthus brevis* (Dowell) Standl	Violaceae	Green violet		M MH	^23^
*Hydrocotyle mexicana* Schltdl. & Cham	Araliaceae	Native pennywort		MH	^23,33^
*Indigofera miniata* Ortega	Leguminosae	Scarlet pea		D	^23,33^
*Justicia spicigera* Schltdl.	Acanthaceae	Mohintli	E		^42,73^
*Lippia umbellata* Cav.	Verbenaceae	Hierba dulce	E		^53^
*Manilkara zapota* (L.) P.Royen	Sapotaceae	Sapodilla		MH	^70^
*Matricaria chamomilla* L.	Compositae	Chamomille		D, MH	^12,22,23,50^
*Melia azedarach* L.	Meliaceae	Chinaberry	E		^42,50^
*Mentha* × *piperita* L.	Lamiaceae	Peppermint		D M MH	^23,33^
*Mentha spicata* L.	Lamiaceae	Spearmint		D	^23,33^
*Origanum vulgare* L.	Lamiaceae	Oregano	A	D	^23,33^
*Phyla scaberrima* (Juss. ex Pers.) Moldenke	Verbenaceae	Aztec sweet herb	E	IP AM	^42,73^
*Plumeria rubra* L.	Apocynaceae	Frangipani alhelí	*A*		^72^
*Pinus sp.*	Pinaceae	Pinus		M MH	^23^
*Persea americana* Mill.	Lauraceae	Avocado	E	D M MH	^23,33,34,50,52,70^
*Piper auritum* Kunth.	Piperaceae	Hoja santa	E		^23,33^
*Pleopeltis polypodioides* (L.) E.G. Andrews & Windham	Polypodiaceae	Resurrection fern	*E*		^53^
*Pluchea odorata* (L.) Cass	Compositae	Marsh fleabane		D	^23,33,52^
*Psittacanthus calyculatus* (DC.) G.Don	Loranthaceae	Injerto de huizache		M MH	^23^
*Psidium guajava* L.	Myrtaceae	Guava		D	^50,51^
*Rosmarinus officinalis* L.	Lamiaceae	Rosemary	E	D	^22,50,52,53^
*Ruta graveolens* L.	Rutaceae	Salab	E A	NS	^22,34^
*Rudbeckia laciniata* L.	Compositae	Dormilón	E		^42,73^
*Salvia microphylla* Kunth	Lamiaceae	Baby sage		D	^75^
*Sambucus canadensis* L.	Adoxaceae	American elder, elderberry		MH	^23^
*Selaginella pallescens* (C. Presl) Spring	Selaginellaceae	Moss fern		MH	^23^
*Solanum nudum* Dunal	Solanaceae	Forest nightshade		MH	^70^
*Struthanthus quercicola* (Schltdl. & Cham.) D.Don	Loranthaceae	Seca palo		MH	^23^
*Styrax argenteus* C.Presl.	Styracaceae	Styrax	E A		^23,33^
*Tagetes lucida* Cav.	Compositae	Day flower	E		^23,33^
*Tagetes micrantha* Cav.	Compositae	Licorice marigold		D	^50^
*Tanacetum parthenium* (L.) Sch.Bip	Compositae	Feverfew, Santamaría	E, A	D	^33,50,72,74^
*Tillandsia recurvata* (L.) L.	Bromeliaceae	Small ballmoss		MH	^70^
*Turnera diffusa* Willd ex Schultz	Passifloraceae	Damania, oreganillo		MD	^76^
*Zaluzania triloba* (Ortega) Pers.	Compositae	Hierba amarga	A		^42,73,74^
*Zea mays* L.	Poaceae	Corn		MH	^23,33^

*A* abortifacient, *E* Emenagogue, *MH* Menstrual hemmorage, *D* dysmenorrhea, NS. Non specified, *M* menorrhagia, *IP* induces menstrual period, *MD* Menstrual disorders unspecified.

**Table 2:** Medicinal plants used in Mexican traditional medicine for conception, pregnancy, delivery, post-partum care, menopause and uterine prolapse.

Scientific name	Common name	Family	Uses	References
*Adiantum poiretii* Wikstr.	Yaru chaqui	Pteridaceae	Postpartum care	^23,33^
*Adiantum tenerum* Sw.	Brittle maidenhair fern	Pteridaceae	Postpartum care	^70^
*Agave atrovirens* Karw. ex Salm-Dyck	Maguey peluquero	Asparagaceae	Postpartum care	^74^
*Ageratina ligustrina* (DC.) R.M.King & H.Rob	Privet-leaved ageratina	Compositae	Postpartum care	^23,33^
*Allium sativum* L.	Garlic	Amaryllidaceae	Oxytocic/Induces labor/Speeds birth	^23^
*Aloe vera* (L.) Burm.f	Aloe	Xanthorrhoeaceae	Unspecified Obstetric problems	^50^
*Anoda cristata* (L.) Schltdl	Crested anoda	Malvaceae	Treats infertility	^33^
*Arbutus xalapensis Kunth*	Texas madrone	Ericaceae	Postpartum care	^23,33^
*Arnica montana* L	Mountain arnica	Compositae	Unspecified Obstetric problems	^50^
*Arundo donax* L.	Cana-brava	Poaceae	Postpartum care	^23,70^
*Baccharis salicina* Torr. & A.Gray	Pineable	Compositae	Oxytocic/Induces labor/Speeds birth; Treats infertility; Prevents miscarriages	^23^
*Bidens pilosa* L.	Black-jack	Compositae	Promotes fertility	^52^
*Brickellia* *secundiflora* (Lag.) A.Gray	Jara blanca	Compositae	Postpartum care; Treats infertility; Prevents miscarriage	^23,33^
*Buddleja cordata* Kunth	Tepozán	Scrophulariaceae	Postpartum care	^53^
*Buxus sempervirens* L.	Boxwood	Buxaceae	Postpartum care	^53^
*Byrsonima crassifolia* (L.) Kunth	Golden spoon	Malpighiaceae	Inflamed uterus	^22^
*Calea ternifolia* Kunth	Zacatechichi	Asteraceae Bercht. & J.Presl	Postpartum care	^52^
*Chenopodium incisum* Poir	Goosefoot	Amaranthaceae	Oxytocic/Induces labor/Speeds birth	^53^
*Chromolaena odorata* (L.) R.M.King & H.Rob	Siam weed	Compositae	Postpartum care	^52^
*Chrysactinia mexicana* A.Gray	Damianita daisy	Asteraceae	Promotes fertility	^77^
*Citrus aurantiifolia* (Christm.) Swingle	Key lime	Rutaceae	Contraceptive	^33^
*Clidemia setosa* (Triana) Gleason	Santa Marta	Melastomataceae	Treats infertility	^33^
*Cocos nucifera* L.	Coconut palm	Arecaceae	Stops hemorrhage during labor	^34^
*Critonia quadrangularis* (DC.) R.M.King & H.Rob	Tabaquillo	Compositae	Postpartum care	^23,33^
*Crescentia cujete* L.	Cujete	Bignoniaceae	Oxytocic/Induces labor/Speeds birth	^23,33^
*Cuminum cyminum* L.	Cumin	Apiaceae	Oxytocic/Induces labor/Speeds birth	^33^
*Desmodium incanum* DC	Zarzabacoa comun	Fabaceae	Antiabortifacient	^22^
*Dioscorea composita* Hemsl.	Barbasco	Dioscoreaceae	Contraceptive	^42,73^
*Dodonaea viscosa* (L.) Jacq.	Native hop bush	Sapindaceae	Postpartum care; Spasmolytic activity; Treats infertility; Prevents miscarriage	^23,33^
*Dysphania ambrosioides* (L.) Mosyakin & Clemants	Epazote	Amaranthaceae	Oxytocic/Induces labor/Speeds birth	^23,33^
*Epiphyllum crenatum* (Lindl.) Don	Climbing orchid cactus	Cactaceae	Anti-abortifacient	^22^
*Erigeron karvinskianus* DC.	Mexican fleabane	Asteraceae Bercht. & J.Presl	Osteoporosis	^12,50^
*Erythrina caribaea* Krukoff & Barneby	Coral-tree	Leguminosae	Oxytocic	^34^
*Euphorbia pulcherrima* Willd. ex Klotzsch	Christmas-flower	Euphorbiaceae	Contraceptive	^23^
*Euphorbia tithymaloides* L.	Red bird flower	Euphorbiaceae	Uterine prolapse	^70^
*Eysenhardtia polystachya* (Ortega) Sarg	Kidney-wood	Leguminosae	Contraceptive	^23^
*Fleischmannia pycnocephala* (Less.) R.M.King & H.Rob	Flor de octubre	Compositae	Postpartum care	^52^
*Foeniculum vulgare* Mill.	*Fern*	Apiaceae	Breast feeding, menopause	^53^
*Gaultheria acuminata* Schltdl. & Cham	*Wintergreen*	Ericaceae	Postpartum care; treat infertility; Prevents miscarriage	^23,33^
*Guazuma ulmifolia* Lam.	West Indian Elm	Malvaceae	Expel placenta	^22^
*Helianthemum glomeratum* (Lag.) Lag. ex Dunal	Damiana	Cistaceae	Osteoporosis	^12,50^
*Heliocarpus glanduliferus* B.L. Rob. Ex Rose	–	Malvaceae	Oxytocic/Induce labor/Speed delivery	
*Heliocarpus mexicanus* (Turcz.) Sprague	Majaguillo	Malvaceae	Oxytocic/Induce labor/Speed delivery	^70^
*Hibiscus rosa-sinensis* L.	Chinese hibiscus	Malvaceae	Menopause	^70^
*Hybanthus attenuatus* (Humb. & Bonpl. ex Schult.) Schulze-Menz	Western Green violet	Violaceae	Sterilizer	^70^
*Hydrocotyle mexicana* Schltdl. & Cham	Native pennywort	Araliaceae	Postpartum care	^23,33^
*Hylocereus undatus (Haw.)* Britton & Rose	Night-blooming ceresus	Cactaceae	Postpartum care	^70^
*Iostephane trilobata* Hemsl.	Hierba del manso	Compositae	Postpartum care; treats infertility; speeds birth	^23,33^
*Lippia alba* (Mill.) N.E.Br. ex Britton & P.Wilson	Bushy Matgrass	Verbenaceae	Postpartum care	^12,23,33^
*Liquidambar styraciflua* L.	Sweet gum	Altingiaceae	Obstetric problems	^75^
*Litsea glaucescens* Kunth	Mexican bay leaf	Lauraceae	Postpartum care; treats infertility	^33^
*Loeselia mexicana* (Lam.) Brand	Mexican false calico	Polemoniaceae	Postpartum care	^52,53^
*Matricaria chamomilla* L.	Chamomile	Compositae	Oxytocic/induces labor/speeds birth; insomnia; reduce stress	^12,22,23,33,50^
*Melia azedarach* L.	Chinaberry	Meliaceae	Venereal diseases	^42,73^
*Mentzelia aspera L.*	Tropical stickleaf	Loasaceae	Uterine prolapse	^70^
*Miconia argentea* (Sw.) DC.	Capirote	Melastomataceae	Oxytocic/induces labor/speeds birth	^22^
*Mimosa albida* Willd.	Mimosa	Leguminosae	Oxytocic/induces labor/speeds birth;treats infertility	^23,33,52^
*Montanoa tomentosa* Cerv.	Zoapatle	Compositae	Oxytocic/induces labor/speeds birth	^23,33,53^
*Musa sp*	Plantain/Banana	Musaceae	Postpartum care	^70^
*Nectandra sp.*		Lauraceae	Oxytocic/induce labor/speed delivery	^70^
*Nopalea cochenillifera* (L.) Salm-Dyck	Cochineal cactus	Cactaceae	Postpartum care	^70^
*Ocimum basilicum* L.	Sweet basil	Lamiaceae	Labor pains; uterine prolapse	^50,70^
*Peperomia granulosa* Trel.	Baby rubber plant	Piperaceae	Breastfeeding	^34^
*Phragmites australis* (Cav.) Trin. ex Steud	Phragmites communis	Poaceae	Postpartum care	^33^
*Pimenta* dioica (L.) Merr.	Allspice	Murtaceae	Inflamed uterus	^22^
*Piper sanctum* (Miq.) Schltdl. ex C.DC.	Hierba santa	Piperaceae	Postpartum care	^34^
*Piper auritum* Kunth	Hoja Santa	Piperaceae	Oxytocic/induces labor/speeds birth	^23,33^
*Piper umbellatum* L.	Cordoncillo	Piperaceae	Oxytocic/induces labor/speeds birth; uterine prolapse	^33,70,78^
*Pleurothallis cardiothallis* Rchb.f	The Heart Shaped Growth Pleurothallis	Orchidaceae	Treats infertility	^33^
*Pluchea odorata* (L.) Cass	Marsh Fleabane	Compositae	Postpartum care	^23,33,52^
*Podocarpus matudae* Lundell	Palmilla	Podocarpaceae	Contraceptive	^23,33^
*Pouteria sapota* (Jacq.) H. E. Moore et Stearn	Mamey Sapote	Sapotaceae	Uterine prolapse	^22^
*Priva lappulacea* (L.) Pers.	Mozote	Verbenaceae	Prevents miscarriage	^70^
*Pseudobombax ellipticum* (Kunth) Dugand	Xiloxochitl	Malvaceae	Induces sterilization	^42,70,73^
*Psittacanthus calyculatus* (DC.) G.Don	Injerto de huizache	Loranthaceae	Treats infertility; prevents miscarriage	^23,33^
*Punica granatum* L.	Pomegranate	Lythraceae	Stops hemorrhage during labor	^52^
*Quercus elliptica* Née	Encino blanco	Fagaceae	Postpartum care	^23,33^
*Quercus oleoides* Schltdl. & Cham	Encino	Fagaceae	Inflamed uterus	^22^
*Rhynchosia pyramidalis* (Lam.) Urb.	Virility-vine	Fabaceae	Contraceptive	^22^
*Ricinus communis* L.	Castor oil plant	Euphorbiaceae	Treats infertility	^23,33^
*Rosa chinensis* Jacq.	China rose	Rosaceae	Menopause	^53^
*Rosmarinus officinalis* L.	Rosemary	Lamiaceae	Postpartum care; treat vaginal infections	^22,50,52,53^
*Russelia sarmentosa* Jacq.	Russelia sarmentosa	Plantaginaceae	Contraceptive	^23,33^
*Ruta chalepensis* L.	Rue	Rutaceae	Oxytocic/induces labor/speeds birth	^23,33,52,72,74^
*Ruellia sp.*	Cuaimate	Acanthaceae	Postpartum care	^42,73^
*Salix bonplandiana* Kunth	Bonpland willow	Salicaceae	Postpartum care	^52^
*Salix humboldtiana* Willd	Humboldt’s willow	Salicaceae	Postpartum care	^52^
*Sambucus nigra* L.	Eldelberry	Adoxaceae	Obstetric problems	^75^
*Selaginella pallescens* (C. Presl) Spring	Moss fern	Selaginellaceae	Postpartum care	^23^
*Sesamum indicum* L.	Sesame	Pedaliaceae	Breastfeeding	^34^
*Solanum nudum* Dunal	Forest nightshade	Solanaceae	Not stated	^70^
*Styrax argenteus* C.Presl	Styrax	Styracaceae	Contraceptive; oxytocic/induces labor/speeds birth	^23^
*Tagetes erecta* L.	Amapola	Compositae	Postpartum care	^70^
*Tagetes lucida* Cav.	Dayflower	Compositae	Oxytocic/induces labor/speeds birth	^23,33^
*Tecoma stans* (L.) Juss. ex Kunth	Trumpet bush	Bignoniaceae	Postpartum care	^12^
*Tectaria sp*	–	Tectariaceae	Postpartum care	^70^
*Thelypteris kunthii* (Desv.) C.V. Morton	Fern (Helecho macho)	Thelypteridaceae	Contraceptive	^42,73^
*Thelypteris tetragona* (Sw.) Small	Freetip maiden fern	Thelypteridaceae	Postpartum care	^70^
*Tonduzia longifolia* (A.DC.) Markgr.	Palo blanco	Apocynaceae	Postpartum care	^52^
*Turbina corymbosa* (L.) Raf.	Ololiuhqui	Convolvulaceae	Oxytocic/induces labor/speeds birth	^23,33^
*Vanilla planifolia* Jacks. ex Andrews	Vanilla	Orchidaceae	Menopause	^34^
*Zea mays* L.	Corn	Poaceae	Postpartum care; treats infertility	^23,33^
*Zingiber officinale* Roscoe	Ginger	Zingiberaceae	Nausea and vomiting; obstetric problems	^50^

Smith-Oka^70,78^ reported on the use of medicinal plants by the Nahua women in Northern Veracruz, Mexico. The results of these studies showed that approximately 80 plant species were used medicinally, and women had the most knowledge about medicinal plants, particularly married women with children. Twenty-six of the plant species collected were used to treat reproductive health issues, including contraception, conception, menstruation, pregnancy, and the postpartum period. Five medicinal herbs were used to increase conception, foster pregnancy, or contraception. *Priva lappulacea* (L.) Pers. was a plant used to prevent miscarriages^69,70,78^. *Hamelia patens* Jacq. [syn. *Hamelia erecta*] and *Bombax ellipticum* Kunth (Malvaceae) were combined and ingested as a tea to induce complete sterility in women (Table 1). Another plant, *Cydista potosina* was prepared as a tea and used for the same purpose, while the use of a preparation of *Hybanthus attenuatus* (Humb. & Bonpl. ex Schult.) Schulze-Menz (Malpighiales) was reported to facilitate conception. Five plants were used by the Natau women for treating heavy menstruation, specifically *Tillandsia recurvata* (Bromeliaceae)*, H. patens, Manikara zapota* (L.) P Royen (Sapotaceae)*, Solanum nudum*, and *Persea americana*^69,70,78^. Two plants were administered to speed up the birthing process-namely *Nectandra* sp. (species not specified) and *Heliocarpus glanduliferus* B.L. Rob. Ex Rose (Malvaceae). Four plants were used to treat uterine prolapse, a condition common to these women. A tea of *Mentzelia aspera* L. (Loasaceae) was taken orally, while for the other plants *Ocimum basilicum, Piper umbellatum*, and *Pedilanthus thytimaloides* were used as a steam or vaginal wash. In terms of the postnatal period, seven plants, namely *Arundo donax, Adiantum tenerum, Hylocereus undatus, Nopalea cochenillifera, Tagetes erecta*, *Tectaria* sp., *Musa* sp. and sometimes *Thelypteris tetragona* were combined in water and used to bathe mother and newborn after birth^69,70,78^. In addition, two plants, *Solanum nudum* and *Hibiscus rosa-sinensis*, were used to reduce crying in infants. Interestingly, many of the medicinal plants used by women were cited in pre-Columbian codices, suggesting that their historical use in Mexico exceeded 500 years, and that these medicinal plants were more broadly used across large regions in Mexico^69,70,78^. However, most of these plants have no safety or efficacy data to support their use during pregnancy.

Pérez-Nicolás et al.^75^ investigated the relationship between sociodemographic factors, including age, economic activity, education, socioeconomic levels, gender, and language proficiency with medicinal plant knowledge and use in a Zapotec community in Santiago Camotlán, Sierra Norte of Oaxaca. Analysis of the data showed that 180 species of plants representing more than 60 families were reportedly used in women’s health. Some of these plants were used in MTM for fertility regulation in women, of which numerous medicinal plants were used as emmenagogues and abortifacients. Approximately forty plant species were used for the symptoms of premenstrual syndrome, heavy menstrual bleeding, and dysmenorrhea. Thirty-five species were used for postpartum care and to facilitate breastfeeding, 16 species were used as oxytocic agents to induce labor and speed birth, and six plant species were used to prevent miscarriage. Fourteen plant species were reported to treat infertility or promote fertility, and seven species were used to treat “inflamed” uterus or uterine prolapse^75^. In terms of menopause, three plants species were reported to treat menopause and two plants were used for osteoporosis. These researchers further described several medicinal plants used by mid-wives in Santiago Camotlán, Oaxaca for labor and delivery, as well as childcare. These plant species included *Sambucus nigra* subsp. *canadensis* (L.) Bolli, and *Liquidambar styraciflua* L. (Table 2)^75^.

Our analysis of the data from multiple sources showed that more than 185 species of plants representing over 60 plant families were commonly used in MTM for women’s reproductive health, including menstrual cycle issues, pregnancy, induction of labor, post-partum recovery, menopause and uterine prolapse (Tables 1 and 2). Some plants were used in MTM for fertility regulation in women, of which numerous medicinal plants were used specifically as emmenagogues and abortifacients. Approximately forty plant species were used for the management of the symptoms of premenstrual syndrome, heavy menstrual bleeding, and dysmenorrhea (Table 1). In terms of pregnancy, 35 plant species were used for postpartum care and to facilitate breastfeeding, 16 species were used as oxytocic agents to induce labor and speed birth, and six plant species were used to prevent miscarriage (Table 2). Fourteen plant species were reported to treat infertility or promote fertility, and seven species were used to treat “inflamed” uterus or uterine prolapse. In terms of menopause, three plants species were reported to treat menopause and two plants were used for osteoporosis (Table 2). While there are some experimental and clinical data for some of the more commonly used medicinal plants, many of the plant species have not been scientifically investigated.

## Discussion

Depending on their geographical location within Mexico, it is estimated that between 12 to 60% of Mexican women depend on MTM practices and herbal medicines for their primary healthcare needs^8,51,65–68,70,71,79–83^. The use of MTM is more prevalent in rural areas where access to modern clinics and healthcare is limited, especially during pregnancy. Thus, having access to MTM in these regions is critical for the overall health of these women^8,12,65,68^. MTM is also important to rural indigenous women who are often victims of extreme poverty, gender inequality, ethnic discrimination, and lack the financial resources and authority to seek prenatal and obstetric care^68^. They tend to fear or mistrust Western medical clinics as these clinics and healthcare workers may lack the cultural sensitivity, language, local knowledge and understanding of indigenous traditional medicines needed to provide adequate healthcare^66,82,83^. One study, the Salud Mesoamérica 2015 Initiative, showed that when compared with national averages, the poor, indigenous, and rural patient populations in Mexico and Central America exhibited worse health outcomes^83^. The IMSS and the Mexican Ministry of Health and Welfare operate most of the healthcare clinics, and the lack of staffing of community healthcare workers and physicians, may make the quality of care inconsistent, and not culturally sensitive^79^. Language is also an important aspect of healthcare, and since many Western trained health workers do not speak indigenous languages, indigenous women often have difficulty explaining their symptoms^66,67,83^. Many Mexican women prefer MTM healers, as well as birthing services offered by midwives rather than nurses and doctors, because midwives incorporate traditional beliefs and medicinal plants into their work, thus preserving the connection with their culture. The caveat is that midwives often are not trained for complicated pregnancy and deliveries, which may result in serious complications and death^66–68,79^.

The aim of this investigation was to facilitate the medical recognition of medicinal plants in MTM that have been reported to be used for women's reproductive health conditions by correlating the available clinical data supporting their use. In addition, we have listed other medicinal plants that are also used in MTM for women´s reproductive healthcare that have no clinical data but may serve as a foundation for future scientific and clinical studies. Our review of the medicinal plants and herbal medicines used in MTM showed approximately 185 different species of plants in 60 plant families were used in the management of women’s reproductive disorders. Of these species, 125 were used as emmenagogues and abortifacients, and to treat PMS, heavy menstrual bleeding, dysmenorrhea, and pregnancy. Approximately 16 species were used as oxytocic agents to induce labor and speed birth. Our study differed from previous investigations that focused on specific regions of study or specific areas of women’s health, such as pregnancy^22,23,33,51–53,70,71,74,78^. Our study covered all regions of Mexico for which there were published data and included medicinal plant information covering all women’s reproductive health conditions. Our study had some similarities to those published from Central America such as Guatemala, Costa Rica, and Belize where women (particularly indigenous and rural) are also dependent on plant-based medicines for a wide range of reproductive cycle issues including PMS, pregnancy, and menopause^18,19,65–67,84^. For example, in Guatemala and Costa Rica, *Pimenta diocia, Piper sanctum, Piper auritum, Hibiscus rosa-sinensis, Zingiber officinale* and *Citrus aurantium* are also used for the management of PMS, pregnancy and menopause^18,19,65–67,82^. In Guatemala, *Zingiber officinale* rhizomes are used to prepare a tea that is taken to reduce hot flashes and night sweats; while a tea made from the leaves and flowers of *Hibiscus rosa-sinensis* is used to reduce sweating and treat nervous conditions^18,19^. Furthermore, chamomile (*Matricaria chamomilla*) tea is used to treat nervousness and insomnia and bitter orange (*Citrus aurantium*) is used in a bath to relieve night sweats and insomnia^18,19^. The wide-spread use of these plants throughout Mexico and Central America for similar purposes is suggestive of efficacy.

Unlike previous studies of medicinal plants used in MTM for women’s reproductive health, this study correlated data from commonly used medicinal plants with available published clinical data supporting their use. While many of the cited plant species have little experimental pharmacological support for their traditional uses, for some of the more commonly used plant species there was considerable amount of supportive clinical and experimental data. Particularly medicinal plants that are used in other countries for similar purposes, such as ginger, chamomile and fennel. Extracts of these plants are commonly used by women in Europe, the Middle East and China for the management of anxiety, pregnancy, PCOS and the symptoms of PMS^24–29^. Many of the clinical trials for these herbal medicines were performed in the Middle East and the USA showing continued global interest in medicinal plants to treat a wide range of conditions including PCOS, PMS, menopause, and General Anxiety Disorders^24–29,32–36^. Some of the limitations of this study include the fact that it relies on data obtained from secondary sources such as peer-reviewed journals, books, and databases, and not from direct field work. Thus, our study relies on the integrity of previously published researcher’s methodology, and their interpretation of the results. Furthermore, MTM is a complex healthcare system that incorporates many aspects of religion, spirituality, and culture, that were not included in the current work.

While Mexican women regularly use herbal medicines and medicinal plants to treat women’s reproductive health issues including pregnancy, for most of these plant species there is little pharmacological data to support their safety and efficacy. Since pregnancy appears to be one of the more common uses of medicinal plants in MTM, it is critical that at least basic studies should be performed to validate at least the safety of these medicinal plants. Other issues posed include the use of many plant species in MTM to treat amenorrhea or those directly used as an abortifacient, indicating that these medicinal plants may have direct effects on the uterus and ovaries, or may exert toxic effects in women or the fetus. Therefore, it is essential that some in vivo scientific investigations be performed to assess toxicity.

The highest prevalence of MTM use appears to occur in the states of Chiapas, Morelos, Oaxaca, Veracruz, Guerrero, Chihuahua, Yucatan, and Veracruz, where the use of MTM and medicinal plants for the management of reproductive health issues by women appears to be extensive. Considering the population of the country is > 128 million and women make up 51.08%, it is essential that more research be performed to assess the safety and efficacy of the medicinal plants currently being used, especially during pregnancy. Fortunately, within Mexico there are numerous private and public institutions that are responsible for the preservation and development of traditional medicine, including the National Institute of Indigenous Peoples (Instituto Nacional de los Pueblos Indígenas- INPI), the Mexican Social Security Institute—Solidarity (Instituto Mexicano del Seguro Social- IMSS/Solidaridad), and the National Council of Traditional Indigenous Medicine Men (Consejo Nacional de Médicos Indígenas Tradicionales—CONAMIT)^8^. Some of these institutions work together to prevent the loss of MTM information. The IMSS/Solidaridad works to promote healthcare in rural/indigenous populations in conjunction with allopathic and Indigenous doctors, traditional healers, and midwives; to develop and maintain medicinal gardens; and to protect traditional healing methods, as well as prevent disease. The IMSS-Solidarity also offers training to health service personnel in the form of workshops on traditional medicine practices, medical anthropology, and other topics. CONAMIT’s responsibility is to legislate MTM^8^.

## Conclusions

Currently, Mexican traditional medicine is a holistic system that combines the ancient healing traditions of the Maya and Aztecs, with that of Spanish and African practices. MTM is essential for healthcare in Mexico and has survived conquest, colonization, and the introduction of Western medicine practices because many MTMs require little if any equipment, are easily transported and obtained. MTM information has been passed down to each generation orally, most often without documentation, although documentation has been increasing over the last 50 years. Although some commonly used medicinal plants and practices in MTM have Asian, European, or Middle Eastern roots, they were incorporated into indigenous Mexican healing practices during colonization and continue to be used today. As in most countries, Mexican women play a central role in family healthcare and are traditionally the providers of medical care and the transmitters of medical knowledge and beliefs. Thus, they are critical for the health of the entire community, particularly the children, all of whom are taken care of exclusively by their female family members. Complications to women’s health are attitudes that prevent women from seeking modern medical services for themselves or their children, due to cultural and religious practices within indigenous communities. Gender inequality is still a serious problem in all countries, including Mexico^65,68^, where many women are poor and lack the financial resources and authority to seek healthcare. In this work, we have shown that medicinal plants in MTM play an essential role in the provision of culturally sensitive and accessible healthcare for the management of women’s reproductive health conditions. Plant-based medicines are used for all aspects of reproductive health including the management of menstrual cycles, PMS, pregnancy, labor, and delivery, as well as menopause. Interestingly, while many plants have no supporting experimental pharmacology, some of the more commonly used plants have significant clinical and experimental data to support their use. Thus, for those medicinal plants with no experimental support, the data presented here may be used as a foundation for future research investigations.

Mexican traditional medicine and associated medicinal plants provide culturally appropriate healthcare interventions that are accessible, inexpensive and, where clinically validated for safety and efficacy, may be integrated into Western clinical care. While some medicinal plants have been scientifically investigated, many MTM medicinal plants, including those used during pregnancy have not been extensively studied for safety and efficacy. Considering the critical role that MTM plays in the health of women and their families, research and development of these practices and medicinal plants is essential to validate their safety and efficacy and should be used as the basis for future research. Continued scientific investigations into Mexican traditional medicines will make these products safer, improve healthcare initiatives and allow for the development of industry and accessibility for MTM medicinal plant products.

## Data Availability

All data and cited studies are peer-revied journal articled, preprints and books available in the public domain; some may require relevant database or journal subscriptions for access. All data used for the foundation of the manuscript are included in the article and may be obtained by contacting the corresponding author at: mahady@uic.edu.
